# Time to surgery and myo-d expression in biceps muscle of adult brachial plexus injury: a preliminary study

**DOI:** 10.1186/s13104-023-06317-y

**Published:** 2023-04-13

**Authors:** Heri Suroto, Gestana Retaha Wardana, Julius Albert Sugianto, Dina Aprilya, Steven Samijo

**Affiliations:** 1 Department of Orthopaedic & Traumatology, Faculty of Medicine, Universitas Airlangga/Dr. Soetomo General Hospital, Surabaya, 60132 Indonesia; 2 Cell and Tissue Bank-Regenerative Medicine, Faculty of Medicine, Dr Soetomo General Academic Hospital, Universitas Airlangga, Surabaya, 60132 Indonesia; 3 Orthopedic and Traumatology Department, Siloam Agora Hospital, Jakarta, Indonesia; 4 Zuyderland Medisch Centrum, Geleen, Netherlands

**Keywords:** Adult brachial plexus injury, Satellite cell, MyoD, Time to surgery

## Abstract

**Background:**

Brachial Plexus Injury (BPI) is one of the peripheral nerve injuries which causes severe functional impairment and disability. Without prompt treatment, prolonged denervation will cause severe muscle atrophy. MyoD, which is expressed by satellite cells, is one of the parameters that relate to the regeneration process in post-injury muscle and it is presumed to determine the clinical outcome following neurotization procedure. This study aims to understand the correlation between time to surgery (TTS) and MyoD expression in satellite cells in the biceps muscle of adult brachial plexus injury patients.

**Methods:**

Analytic observational study with a cross-sectional design was conducted at Dr. Soetomo General Hospital. All patients with BPI who underwent surgery between May 2013 and December 2015 were included. Muscle biopsy was taken and stained using immunohistochemistry for MyoD expression. Pearson correlation test was used to assess the correlation between MyoD expression with TTS and with age.

**Results:**

Twenty-two biceps muscle samples were examined. Most patients are males (81.8%) with an average age of 25.5 years. MyoD expression was found to be highest at TTS of 4 months and then dropped significantly (and plateau) from 9 to 36 months. MyoD expression is significantly correlated with TTS (r=-0.895; p = 0.00) but not with age (r=-0.294; p = 0.184).

**Conclusion:**

Our study found, from the cellular point of view, that treatment of BPI needs to be done as early as possible before the regenerative potential - as indicated by MyoD expression – declined.

**Supplementary Information:**

The online version contains supplementary material available at 10.1186/s13104-023-06317-y.

## Introduction

Brachial Plexus Injury (BPI) is the most disabling form of peripheral nerve injury which causes severe functional impairment and poor quality of life [1]. It is estimated to happen in 1.2% of patients with multiple traumatic injuries with an incidence rate of 1.64 cases out of 100.000 people [2]. The impact of BPI is not only on the nerves of the brachial plexus but also on the denervated muscle. Prolonged denervation of the effector’s muscle has various impacts on muscle and worsens the longer it is denervated [3]. Denervated muscle will undergo atrophic changes in a biphasic manner: rapid muscle mass loss in the first 2 weeks and more gradually afterwards [3–5]. At the 3rd month, a decrease in fiber diameter, intramuscular fibrosis, and reduction of motor endplates can be observed [6, 7]. Therefore, management in a timely manner is needed to avoid this worst possible outcome [1].

The timing of surgery for traumatic BPI remains controversial [1]. Elucidating the best time to repair a traumatic BPI can be done by assessing its regenerative potential preoperatively or its functional outcome postoperatively. This study aims to assess the first: by assessing the correlation between time to surgery and MyoD expression which has an important role in muscle regeneration. To the author’s knowledge, similar studies assessing MyoD expression in human after a denervating injury, especially brachial plexus injury, is rare. Most studies are either in vitro or in vivo [8–11]. While studies in human are mostly on its effect after exercise or in its relation to sarcopenia [12–15]. Therefore, this is the first study on MyoD expression in human after muscle denervation in traumatic BPI patients.

## Methods

### Data extraction and ethics

An analytical observational study with a cross-sectional design was conducted. The study protocol was reviewed and approved by Dr. Soetomo General Hospital’s Ethical Committee (No.647/Panke.KKE/XI/2016). Consent for publication is not applicable as the sample used is part of routine procedure. The population of the study is all patients with brachial plexus injury who underwent surgery, either by nerve procedure, muscle procedure, or both, in Dr. Soetomo General Hospital, Surabaya, East Java, Indonesia between May 2013 and December 2015. These patients are then screened based on our criteria: inclusion criteria are: (1) Patients who had BPI caused by a trauma (2) patients with no other disease that might affect nerve and muscle and (3) patients who gave consent to be research subject; exclusion criteria are: (1) muscle samples that cannot be evaluated. Written informed consent has been obtained from each patient before the surgery and data collection. Data of sex, age, time to surgery (TTS), anatomic location of the injury, type of injury, muscle strength before surgery (using Medical Research Council Scale), and amount of MyoD expressing satellite cells from each sample were collected. Time to surgery is defined as the time between the incidence of brachial plexus injury and the day of surgery. Anatomic location of injury was based on surgical aspect of the injury whether it is upper type, lower type or total type lesion and whether it is pre or post ganglionic. Sample preparation and immunohistochemistry staining procedure is described at the appendix A.

### Data analysis

Compiled data were analyzed descriptively and presented as tables. Statistical analysis on the relationship between MyoD expressing cells with TTS and patient’s age was done using SPSS version 21. The normality data test was firstly conducted using Shapiro Wilk and, if the data is normally distributed, the correlation was tested using the Pearson test. Values of p < 0.05 are considered significant. A Scatter plot is also drawn to estimate the correlation between the two.

## Result

**Fig. 1:**
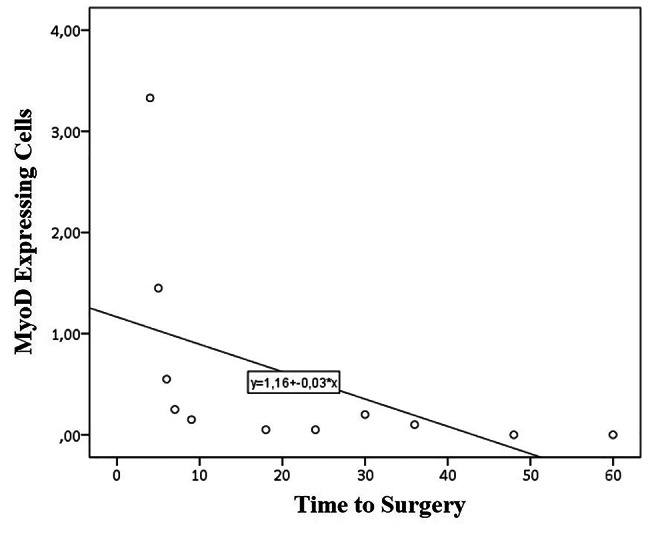
Scatter Plot correlating MyoD expressing cells and time to surgery. [Personal Documentation]

**Fig. 2:**
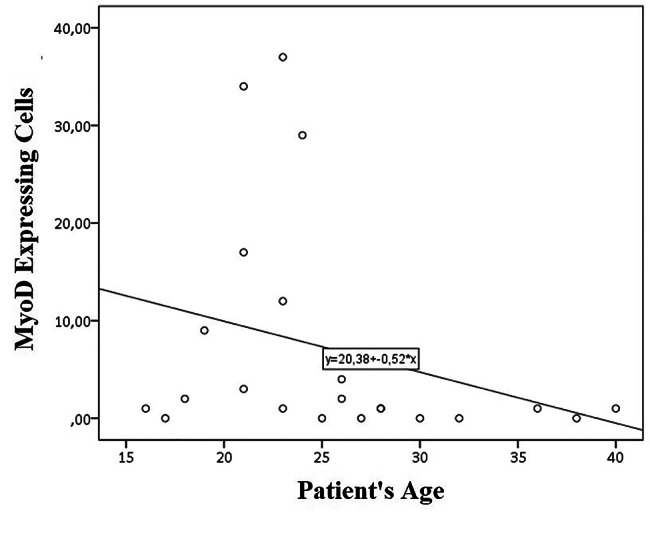
Scatter Plot correlating MyoD expressing cells and patient’s age. [Personal Documentation]

Twenty-two biceps muscle samples were examined. Demographic data of all samples are shown in Table 1. Most patients are males (81.8%) with an average age of 25.5 ± 6.54 years. Time to surgery spans from 4 to 60 months with an average of 21 months. Injury types are mostly complete post-ganglion type (50%) and most patient had a very low motoric strength on bicep abduction (1,27 ± 0,46) The complete data is tabulated at appendix B. The result is then grouped based on the time to surgery (Table 2; Fig. 1) and on patient’s age (Fig. 2).

**Table 1:** Demographic data of brachial plexus injury patients

Patient	Sex	Age (year)	TTS (month)	Muscle Strength	Anatomy Injury
1	M	21	4	1	C5-6-7 postganglion
2	F	24	4	1	C5-T1 postganglion
3	F	23	4	2	C5-T1 postganglion
4	M	23	5	1	C5-6 postganglion, C7-T1 preganglion
5	F	21	5	1	C5-6 postganglion, C7-T1 preganglion
6	M	19	6	1	C5-6 postganglion, C7-T1 preganglion
7	M	18	6	2	C5-T1 postganglion
8	M	26	7	1	C5-6 postganglion, C7-T1 preganglion
9	M	28	7	2	C5-T1 postganglion
10	M	26	9	1	C5-6 postganglion, C7-T1 preganglion
11	M	40	9	1	C5-6-7 postganglion
12	M	27	18	1	C5-T1 postganglion
13	M	23	18	2	C5-T1 postganglion
14	M	17	24	1	C5-6 postganglion, C7-T1 preganglion
15	F	36	24	1	C5-T1 postganglion
16	F	16	30	1	C5-T1 postganglion
17	M	21	30	1	C5-T1 postganglion
18	M	28	36	2	C5-6 postganglion, C7-T1 preganglion
19	F	25	48	2	C5-6 postganglion, C7-T1 preganglion
20	M	30	48	1	C5-T1 postganglion
21	M	32	60	1	C5-6-7 postganglion
22	M	38	60	1	C5-T1 postganglion
**Average**		25.55 ± 6.54	21 ± 18.81	1,27 ± 0,46	

**Table 2:** Satellite cells expressing MyoD based on TTS

TTS	N	Cell expression	Satellite Cells Expressing MyoD
4 months	3	10.00	3.33
5 months	2	2.90	1.45
6 months	2	1.10	0.55
7 months	2	0.50	0.25
9 months	2	0.30	0.15
18 months	2	0.10	0.05
24 months	2	0.10	0.05
30 months	2	0.40	0.20
36 months	1	0.10	0.10
48 months	2	0.00	0.00
60 months	2	0.00	0.00

**Fig. 3:**
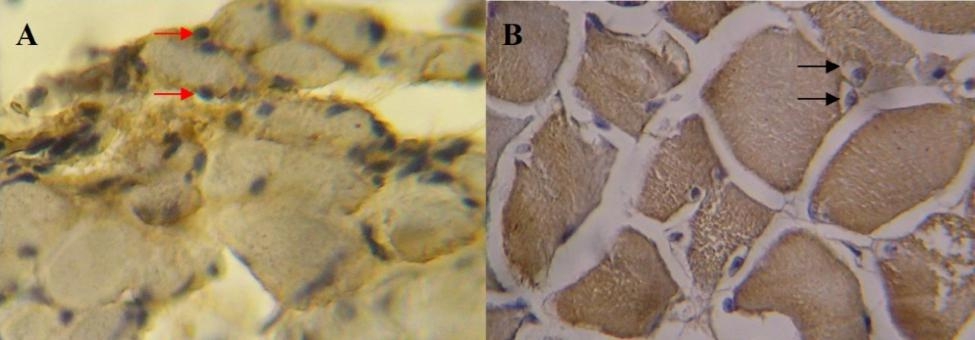
Cross sectional image of biceps muscle (400x magnification). From patient with TTS of 4 months, MyoD expressing satellite cells are marked by the red arrow (A). From patient with TTS of 9 months, most satellite cells do not express MyoD, satellite cells are marked with black arrow (B). [Personal Documentation]

Photograph of the immunohistochemical result is as shown on Fig. 3. As displayed on Fig. 1, MyoD expression is highest at TTS of 4 months which then dropped significantly, plunges to near zero 6 months after injury, and plateau from 9 to 36 months. No cells express MyoD at 48–60 months. In its relation with age, MyoD showed an inverse relation: decreasing MyoD expression on aged population compared to younger one. On statistical analysis, MyoD expression is found to be significantly correlated with TTS (r=-0.895; p = 0,00) but not with age (r=-0.294; p = 0.184).

## Discussion

BPI causes defects to the muscle and nerves of the upper extremity. The nerve may have different levels of damage, from neuropraxia, axonotmesis, and neurotmesis. The distal part of the injured nerve will also undergo resorption by phagocytes (Wallerian degeneration). The denervated target organ (muscle) will atrophy and if no reinnervation occurs in 2 years, the damage will be permanent [16].

The best time of surgery for traumatic BPI has been subject of debates over the years, with a move towards early intervention. Although, there is an accepted time frame of 12 to 18 months for muscle reinnervation to occur after neurotization before the irreversible motor end plate degeneration occurs, there is no definite evidence to support this. Other factors are thought to have important roles in determining the clinical outcome. A combination of slow axonal regeneration, structural changes in muscle targets, and an increasingly less supportive stromal environment for regeneration are believed to contribute to a poor functional recovery in late phases [17].

Terzis et al. [18] stated that the most important factor to determine the clinical outcome in BPI surgery is denervation time, which is the time between the injury and the surgery. Bentolila et al. [19] stated that BPI surgery, specifically nerve procedure (nerve grafting) is best done before the 6th month. After the 6th month, there will be muscle atrophy, fibrosis, and joint stiffness which may hinder the improvement of the clinical outcome after the nerve procedure [20]. Mennen also added that excessive fibrosis in the distal nerve ends in patients that underwent the surgery after 6 months may hinder the muscle regeneration process and will produce a bad clinical outcome post-op [21].

At the point of view of the muscle, Lee et al. [22] study found that muscle fibrosis and atrophy will immediately begin after denervation and plateaus after four months when 60–80% of muscle volume has been lost. Motor endplates actually increase within muscle but functional reinnervation is unlikely beyond 12 months due to the progressive fibrosis. Skeletal muscle regeneration from an atrophy can be achieved through a cascade of intrinsic and extrinsic signals leading the myogenetic process from satellite cells to effective skeletal muscle fiber [23].

Satellite cells are heterogenous cells that is able to do both asymmetric and symmetric division. Asymmetric division is to produce myogenic progenitors while symmetric divisions to multiply satellite cell. Satellite cells are mitotically quiescent and can be activated at times of injury or growth stimulation. Upon activation, satellite cells will mainly co-express a combination of paired box protein Pax7 and Myoblast determination protein1 (MyoD).

The expression of Pax7 and MyoD can be used as an indicator of on which state the satellite cells are in: resting phase, activation to cycling myoblasts, or differentiation to myocytes. In quiescent phase, only Pax7 is expressed and no MyoD is expressed. During its process to become myofibers, expression of Pax7 is decreased gradually and is replaced by MyoD. Once committed to differentiation to myocytes, only MyoD is expressed [23–25]. Other regulatory factors such as Myf5 and MyoG are also expressed during this process but is not mentioned to simplify as it is beyond the scope of this paper [25].

MyoD is one of transcription factors belonging to myogenic regulatory factor family (MRF) which has an important role in myogenic differentiation. MyoD itself was found initially by Davis et al. [26] at 1987 [27]. Uncontrolled expression of MyoD results in depletion of muscle stem cell and premature myogenic differentiation [11, 28]. Due to its importance in myogenic differentiation, MyoD can be used as an indicator of muscle’s regeneration on cellular level. Our study found that MyoD expression is very high on earlier TTS and nearly inexistent 6 months after injury. This indicates that, on cellular point of view, BPI repair would result in better outcome the earlier it is repaired. Initial level of MyoD before repair is important as it is related to muscle’s recovery after rehabilitative exercise [29, 30]. Study by Kosek DJ et al. [30] indicates that lower initial MyoD is related to lower post rehabilitative outcome as expressed by Myofiber’s cross sectional area, type distribution, and type area distribution [30].

This result is also in line with other studies on postoperative function point of view. Timing of BPI surgery usually needs to balance between allowing spontaneous nerve regeneration and preventing denervation atrophy before regeneration is no longer possible [31]. Despite so, a systematic review conducted by Martine E et al. [32] summarized results from 43 studies comprising of 569 patients found that, regardless of BPI injury lesion site, longer TTS and older age results in worse post operative motoric outcome as measured by Medical Research Council muscle grade. The study concludes that generally, a 3-month delay is appropriate for allowing spontaneous nerve regeneration and surgeries are best done in less than 6 months [32].

In its relation with age, our study found that there is no significant relation between MyoD expression and age. Figure 2 does show a spike of MyoD expression on patients aged 20–25 years is noticeable but it is important to note that it is because most samples with TTS of 4–6 months are 20–25 years. Therefore, increase in MyoD expression at that age range is not caused by age per se, but because of shorter TTS. Previous studies found that worse outcomes are associated with older age [18, 32–35]. Some studies found 30 years as a limit to worse outcomes while another at 40 years [33, 35, 18]. Not only does the initial MyoD expression is lower in older people, but MyoD expression is also lower in post rehabilitative setting in older compared to younger people [30]. Higher cortical plasticity in young patients are thought to be a strong factor in outcomes of patient with BPI [36].

Generally, this study found that TTS is related to MyoD expression and this gives information about muscle regeneration potential post denervation indirectly. Our study found that the amount of MyoD expressing satellite cells, an indirect indicator of muscle’s regenerative potential, is significantly correlated with TTS (r=-0.895; p = 0,00) and plunges to near zero 6 months after injury. In its relation with age, MyoD expressing satellite cell is found not correlated (r=-0.294; p = 0.184) with age but this result is not supported with most of other studies.

## Conclusion

The treatment of traumatic BPI has moved toward an earlier neurotization to improve functional outcome. Our study found yet another evidence, on cellular point of view, that before 6 months, nerve procedure in adult patient with traumatic BPI have a more favorable result related to the good muscle regenerative potential.

### Limitations

Several limitations in the making of this study cannot be avoided. The limitations are: (1) no standardized reference regarding how many MyoDs need to be expressed for optimum regeneration potential, (2) no directly comparable study on MyoDs after BPI which can be used to ascertain the result of this study, and (3) small sample size on each group of both TTS and age which might skew the result. Even so, given the low incidence of BPI worldwide, we believe this study is worthy to be published.

## Electronic supplementary material

Below is the link to the electronic supplementary material.

Supplementary Material 1

Supplementary Material 2

## Data Availability

All data generated or analyzed during this study are included in this published article.

## References

[1] Park HR, Lee GS, Kim IS, Chang J-C. Brachial Plexus Injury in Adults. The Nerve [Internet]. 2017 Apr 30;3(1):1–11. Available from: http://thenerve.net/journal/view.php?doi=10.21129/nerve.2017.3.1.1

[2] Smania N, Berto G, La Marchina E, Melotti C, Midiri A, Roncari L (2012). Rehabilitation of brachial plexus injuries in adults and children. Eur J Phys Rehabil Med.

[3] Yang X, Xue P, Chen H, Yuan M, Kang Y, Duscher D et al. Denervation drives skeletal muscle atrophy and induces mitochondrial dysfunction, mitophagy and apoptosis via miR-142a-5p/MFN1 axis. Theranostics [Internet]. 2020;10(3):1415–32. Available from: http://www.thno.org/v10p1415.htm 10.7150/thno.40857 PMC6956801 31938072

[4] O′Leary MFN, Vainshtein A, Carter HN, Zhang Y, Hood DA. Denervation-induced mitochondrial dysfunction and autophagy in skeletal muscle of apoptosis-deficient animals. Am J Physiol Physiol [Internet]. 2012 Aug 15;303(4):C447–54. Available from: https://www.physiology.org/doi/10.1152/ajpcell.00451.2011 10.1152/ajpcell.00451.2011 22673615

[5] Adhihetty PJ, O’Leary MFN, Chabi B, Wicks KL, Hood DA. Effect of denervation on mitochondrially mediated apoptosis in skeletal muscle. J Appl Physiol [Internet]. 2007 Mar;102(3):1143–51. Available from: https://www.physiology.org/doi/10.1152/japplphysiol.00768.2006 10.1152/japplphysiol.00768.2006 17122379

[6] Kobayashi J, Mackinnon SE, Watanabe O, Ball DJ, Ming Gu X, Hunter DA et al. The effect of duration of muscle denervation on functional recovery in the rat model. Muscle Nerve [Internet]. 1997 Jul;20(7):858–66. Available from: https://onlinelibrary.wiley.com/doi/10.1002/(SICI)1097-4598(199707)20:7%3C858::AID-MUS10%3E3.0.CO;2-O 10.1002/(sici)1097-4598(199707)20:7<858::aid-mus10>3.0.co;2-o 9179158

[7] Fu S, Gordon T. Contributing factors to poor functional recovery after delayed nerve repair: prolonged denervation. J Neurosci [Internet]. 1995 May 1;15(5):3886–95. Available from: https://www.jneurosci.org/lookup/doi/10.1523/JNEUROSCI.15-05-03886.1995 10.1523/JNEUROSCI.15-05-03886.1995 PMC6578254 7751953

[8] Jama A, Huang D, Alshudukhi AA, Chrast R, Ren H. Lipin1 is required for skeletal muscle development by regulating MEF2c and MyoD expression. J Physiol [Internet]. 2019 Feb 26;597(3):889–901. Available from: https://onlinelibrary.wiley.com/doi/10.1113/JP276919 10.1113/JP276919 PMC6355634 30511745

[9] Scionti I, Hayashi S, Mouradian S, Girard E, Esteves de Lima J, Morel V et al. LSD1 Controls Timely MyoD Expression via MyoD Core Enhancer Transcription. Cell Rep [Internet]. 2017 Feb;18(8):1996–2006. Available from: https://linkinghub.elsevier.com/retrieve/pii/S2211124717301481 10.1016/j.celrep.2017.01.078 28228264

[10] Luo D, de Morree A, Boutet S, Quach N, Natu V, Rustagi A et al. Deltex2 represses MyoD expression and inhibits myogenic differentiation by acting as a negative regulator of Jmjd1c. Proc Natl Acad Sci [Internet]. 2017 Apr 11;114(15):E3071–80. Available from: http://www.pnas.org/lookup/doi/10.1073/pnas.1613592114 10.1073/pnas.1613592114 PMC5393251 28351977

[11] Lahmann I, Bröhl D, Zyrianova T, Isomura A, Czajkowski MT, Kapoor V et al. Oscillations of MyoD and Hes1 proteins regulate the maintenance of activated muscle stem cells. Genes Dev [Internet]. 2019 May 1;33(9–10):524–35. Available from: http://genesdev.cshlp.org/lookup/doi/10.1101/gad.322818.118 10.1101/gad.322818.118 PMC6499323 30862660

[12] Vogiatzis I, Stratakos G, Simoes DCM, Terzis G, Georgiadou O, Roussos C et al. Effects of rehabilitative exercise on peripheral muscle TNF, IL-6, IGF-I and MyoD expression in patients with COPD. Thorax [Internet]. 2007 May 25;62(11):950–6. Available from: https://thorax.bmj.com/lookup/doi/10.1136/thx.2006.069310 10.1136/thx.2006.069310 PMC2117139 17573449

[13] Vogiatzis I, Simoes DCM, Stratakos G, Kourepini E, Terzis G, Manta P et al. Effect of pulmonary rehabilitation on muscle remodelling in cachectic patients with COPD. Eur Respir J [Internet]. 2010 Aug 1;36(2):301–10. Available from: http://erj.ersjournals.com/cgi/doi/10.1183/09031936.00112909 10.1183/09031936.00112909 20110400

[14] Brzeszczyńska J, Meyer A, McGregor R, Schilb A, Degen S, Tadini V et al. Alterations in the in vitro and in vivo regulation of muscle regeneration in healthy ageing and the influence of sarcopenia. J Cachexia Sarcopenia Muscle [Internet]. 2018 Feb;9(1):93–105. Available from: https://onlinelibrary.wiley.com/doi/10.1002/jcsm.12252 10.1002/jcsm.12252 PMC5803613 29214748

[15] Fochi S, Giuriato G, De Simone T, Gomez-Lira M, Tamburin S, Del Piccolo L et al. Regulation of microRNAs in Satellite Cell Renewal, Muscle Function, Sarcopenia and the Role of Exercise. Int J Mol Sci [Internet]. 2020 Sep 14;21(18):6732. Available from: https://www.mdpi.com/1422-0067/21/18/6732 10.3390/ijms21186732 PMC7555198 32937893

[16] Warwick D, Srinivasan H, Solomon L. Peripheral nerve disorder. In: Arnold H, editor. Apley’s system of Orthopaedics and Fracture. 9th ed. United Kingdom; 2010. pp. 269–80.

[17] Grinsell D, Keating CP. Peripheral nerve reconstruction after injury: a review of clinical and experimental therapies.Biomed Res Int. 2014;2014. 10.1155/2014/698256 PMC4167952 25276813

[18] Terzis JK, Papakonstantinou KC (2000). The surgical treatment of brachial plexus injuries in adults. Plast Reconstr Surg.

[19] Bentolila V, Nizard R, Bizot P, Sedei L (1999). Complete traumatic brachial plexus palsy: treatment and outcome after repair. J Bone J Surg.

[20] Millesi H (1977). Surgical management of brachial plexus injuries. 1977;2:367 – 79. J Hand Surg.

[21] Mennen U (1999). End to side nerve suture-A technique to repair peripheral nerve injury. S Afr Med J.

[22] Park HR, Lee GS, Kim IS, Chang J-C (2017). Brachial plexus injury in adults. The Nerve.

[23] Le Grand F, Rudnicki MA. Skeletal muscle satellite cells and adult myogenesis. Curr Opin Cell Biol [Internet]. 2007 Dec;19(6):628–33. Available from: https://linkinghub.elsevier.com/retrieve/pii/S0955067407001342 10.1016/j.ceb.2007.09.012 PMC2215059 17996437

[24] Zammit PS. Function of the myogenic regulatory factors Myf5, MyoD, Myogenin and MRF4 in skeletal muscle, satellite cells and regenerative myogenesis. Semin Cell Dev Biol [Internet]. 2017 Dec;72:19–32. Available from: https://linkinghub.elsevier.com/retrieve/pii/S108495211730366X 10.1016/j.semcdb.2017.11.011 29127046

[25] Singh K, Dilworth FJ. Differential modulation of cell cycle progression distinguishes members of the myogenic regulatory factor family of transcription factors. FEBS J [Internet]. 2013 Sep;280(17):3991–4003. Available from: https://onlinelibrary.wiley.com/doi/10.1111/febs.12188 10.1111/febs.12188 23419170

[26] Davis RL, Weintraub H, Lassar AB (1987). Expression of a single transfected cDNA converts fibroblasts to myoblasts. Cell.

[27] Davis RL, Weintraub H, Lassar AB. Expression of a single transfected cDNA converts fibroblasts to myoblasts. Cell [Internet]. 1987 Dec;51(6):987–1000. Available from: https://linkinghub.elsevier.com/retrieve/pii/009286748790585X 10.1016/0092-8674(87)90585-x 3690668

[28] Yamamoto M, Legendre NP, Biswas AA, Lawton A, Yamamoto S, Tajbakhsh S et al. Loss of MyoD and Myf5 in Skeletal Muscle Stem Cells Results in Altered Myogenic Programming and Failed Regeneration. Stem Cell Reports [Internet]. 2018 Mar;10(3):956–69. Available from: https://linkinghub.elsevier.com/retrieve/pii/S2213671118300535 10.1016/j.stemcr.2018.01.027 PMC5918368 29478898

[29] Aguiar A, Vechetti-Júnior I, Alves de Souza R, Castan E, Milanezi-Aguiar R, Padovani C et al. Myogenin, MyoD and IGF-I Regulate Muscle Mass but not Fiber-type Conversion during Resistance Training in Rats. Int J Sports Med [Internet]. 2012 Oct 11;34(04):293–301. Available from: http://www.thieme-connect.de/DOI/DOI?10.1055/s-0032-1321895 10.1055/s-0032-1321895 23059557

[30] Kosek DJ, Kim J, Petrella JK, Cross JM, Bamman MM. Efficacy of 3 days/wk resistance training on myofiber hypertrophy and myogenic mechanisms in young vs. older adults. J Appl Physiol [Internet]. 2006 Aug;101(2):531–44. Available from: https://www.physiology.org/doi/10.1152/japplphysiol.01474.2005 10.1152/japplphysiol.01474.2005 16614355

[31] Gutkowska O, Martynkiewicz J, Urban M, Gosk J. Brachial plexus injury after shoulder dislocation: a literature review. Neurosurg Rev [Internet]. 2020 Apr 30;43(2):407–23. Available from: http://link.springer.com/10.1007/s10143-018-1001-x 10.1007/s10143-018-1001-x PMC7186242 29961154

[32] Martin E, Senders JT, DiRisio AC, Smith TR, Broekman MLD (2018). Timing of surgery in traumatic brachial plexus injury: a systematic review. J Neurosurg.

[33] Coulet B, Boretto JG, Lazerges C, Chammas M. A Comparison of Intercostal and Partial Ulnar Nerve Transfers in Restoring Elbow Flexion Following Upper Brachial Plexus Injury (C5-C6 ± C7). J Hand Surg Am [Internet]. 2010 Aug;35(8):1297–303. Available from: https://linkinghub.elsevier.com/retrieve/pii/S0363502310005101 10.1016/j.jhsa.2010.04.025 20638201

[34] El-Gammal TA, Fathi NA. Outcomes of Surgical Treatment of Brachial Plexus Injuries Using Nerve Grafting and Nerve Transfers. J Reconstr Microsurg [Internet]. 2002;18(1):007–16. Available from: http://www.thieme-connect.de/DOI/DOI?10.1055/s-2002-19703 10.1055/s-2002-19703 11917959

[35] Nagano A (1998). Treatment of brachial plexus injury. J Orthop Sci.

[36] Socolovsky M, Malessy M, Lopez D, Guedes F, Flores L. Current concepts in plasticity and nerve transfers: relationship between surgical techniques and outcomes. Neurosurg Focus [Internet]. 2017 Mar;42(3):E13. Available from: https://thejns.org/view/journals/neurosurg-focus/42/3/article-pE13.xml 10.3171/2016.12.FOCUS16431 28245665

