# Supplementary material for: Time to surgery and myo-d expression in biceps muscle of adult brachial plexus injury: a preliminary study
Source: BMC Res Notes. 2023 Apr 13;16:51. doi: 10.1186/s13104-023-06317-y (PMC10103435; doi:10.1186/s13104-023-06317-y)
Supplement: Supplementary file 1 — Supplementary Material 1 [file 13104_2023_6317_MOESM1_ESM.docx]

**Appendix A. Tissue Preparation and Immunohistochemical Staining**

***Muscle biopsy and tissue preparation***

A biceps muscle biopsy of 1x0.5x0.5 cm^3^ was acquired during either nerve or muscle procedure of brachial plexus injury to restore elbow flexion in our center. The sample was then fixated using formaldehyde 10% in the cassette and then sent for histological examination. After fixation, the sample was washed using flowing water for 2 hours. Afterward, the sample was, sequentially, dehydrated, cleared, and embedded. Dehydration was done using 70% ethanol for 4 x 30 minutes, 80% ethanol for 2 x 30 minutes, 96% ethanol for 1 x 30 minutes, and lastly 100% ethanol 100% for 1 x 30 minutes. After dehydration, the clearing was done using Xylol I for 15 minutes and Xylol II until overnight. Embedding was done by removing the muscle tissue sample from the cassette to the paraffin bath. The sample was soaked in paraffin: xylol (1:1) for 30 minutes and, sequentially treated with paraffin I, paraffin II, and paraffin III for 60 minutes. Lastly, we made the tissue to the print of 2 x 2 x 2 cm^3^ that contained paraffin until solidifies overnight.

***Immunohistochemistry of biopsy sections***

The readied sample is then stained. Immunohistochemical staining was started by deparaffinizing the muscle tissue by washing using, sequentially, xylol for 3x5 minutes, 100% ethanol for 5 minutes, 96% ethanol 2x2 minutes, 90% ethanol 2 minutes, 80% ethanol 2 minutes, flowing water for 1 minute, and lastly soaking the sample in aquadest. The deparaffinized muscle tissue is then treated with H2O2 3% for 15 minutes, washed by Phosphate buffer saline (PBS) pH 7,4 for 3x5 minutes, and then placed in slide. The slide is soaked in citric acid fluid and placed in a high-temperature microwave till bubbling. Afterward, the temperature is lowered for 20 minutes to open the epitope of the protein.

The slide is then bound with the primary antibody. The process starts by washing the slide with PBS for 3x5 minutes. Normal Swine Serum is dropped in the slide as a background sniper to bind the nontarget epitope then washed by PBS for 3x5 minutes. The tissue sample slide put in the primary antibody monoclonal: murine antibody monoclonal for anti-MyoD monoclonal antibody. Antibody monoclonal is then dissolved with TRIS-PBS 1:100 (i.e., 100 µL antibody monoclonal used for every 1 cm^2^ tissue). Incubation is performed for 1 hour/ overnight in a moist room.

The slide was then washed by PBS pH 7,4 for 5 minutes 3 times and incubated in secondary antibody monoclonal (anti murine antibody biotinized) for 20 minutes. After that, it was again washed with PBS pH 7.4 for 5 minutes 3 times. HRP (streptavidin peroxidase conjugate) was dropped in the marked immunologic area for 10 minutes. After washing with PBS pH 7.4 for 5 minutes 3 times, we dropped DAB for 5 minutes and then washed the sample with water for 5 minutes. The slide was put in Mayer’s Hematoxylin for 5 minutes and washed with the flowing water for 5 minutes. The slide was then washed with Ethanol 80%, 90%, 96% sequentially for 5 minutes. Finally, the slide was cleared with xylol for 5 minutes 3 times, mounted by putting deck glass, and sealed with polylysine.

Visual examination of MyoD expressing satellite cells was done using Olympus CX-41 microscope with 400x magnification on lighting number 6. Photographs were taken using a digital camera DP-20. Satellite cells that express MyoD were counted on 10 fields and were averaged.
