# Supplementary material for: Time to surgery and myo-d expression in biceps muscle of adult brachial plexus injury: a preliminary study
Source: BMC Res Notes. 2023 Apr 13;16:51. doi: 10.1186/s13104-023-06317-y (PMC10103435; doi:10.1186/s13104-023-06317-y)
Supplement: Supplementary file 2 — Supplementary Material 2 [file 13104_2023_6317_MOESM2_ESM.docx]

**Appendix B.** MyoD expressing satellite cells in each field of each sample

| Sample | MyoD expressing satellite cells in each field | | | | | | | | | | | Average |
| --- | --- | --- | --- | --- | --- | --- | --- | --- | --- | --- | --- | --- |
|  | **1** | **2** | **3** | **4** | **5** | **6** | **7** | **8** | **9** | **10** |  | |
| 1 | 3 | 6 | 2 | 4 | 3 | 3 | 3 | 3 | 5 | 2 | 3.40 | |
| 2 | 5 | 2 | 3 | 3 | 1 | 2 | 4 | 3 | 3 | 3 | 2.90 | |
| 3 | 6 | 5 | 5 | 6 | 2 | 4 | 4 | 3 | 2 | 2 | 3.70 | |
| 4 | 1 | 2 | 2 | 1 | 3 | 1 | 0 | 1 | 0 | 1 | 1.20 | |
| 5 | 0 | 2 | 0 | 1 | 3 | 0 | 2 | 5 | 0 | 4 | 1.70 | |
| 6 | 1 | 1 | 1 | 0 | 1 | 0 | 1 | 2 | 0 | 2 | 0.90 | |
| 7 | 0 | 0 | 0 | 0 | 1 | 1 | 0 | 0 | 0 | 0 | 0.20 | |
| 8 | 1 | 1 | 0 | 0 | 0 | 0 | 2 | 0 | 0 | 0 | 0.40 | |
| 9 | 0 | 0 | 0 | 0 | 1 | 0 | 0 | 0 | 0 | 0 | 0.10 | |
| 10 | 0 | 0 | 1 | 0 | 0 | 0 | 0 | 0 | 1 | 0 | 0.20 | |
| 11 | 0 | 0 | 0 | 0 | 0 | 0 | 0 | 1 | 0 | 0 | 0.10 | |
| 12 | 0 | 0 | 0 | 0 | 0 | 0 | 0 | 0 | 0 | 0 | 0.00 | |
| 13 | 0 | 0 | 1 | 0 | 0 | 0 | 0 | 0 | 0 | 0 | 0.10 | |
| 14 | 0 | 0 | 0 | 0 | 0 | 0 | 0 | 0 | 0 | 0 | 0.00 | |
| 15 | 0 | 0 | 0 | 1 | 0 | 0 | 0 | 0 | 0 | 0 | 0.10 | |
| 16 | 0 | 0 | 0 | 0 | 0 | 1 | 0 | 0 | 0 | 0 | 0.10 | |
| 17 | 0 | 1 | 0 | 0 | 0 | 0 | 0 | 2 | 0 | 0 | 0.30 | |
| 18 | 1 | 0 | 0 | 0 | 0 | 0 | 0 | 0 | 0 | 0 | 0.10 | |
| 19 | 0 | 0 | 0 | 0 | 0 | 0 | 0 | 0 | 0 | 0 | 0.00 | |
| 20 | 0 | 0 | 0 | 0 | 0 | 0 | 0 | 0 | 0 | 0 | 0.00 | |
| 21 | 0 | 0 | 0 | 0 | 0 | 0 | 0 | 0 | 0 | 0 | 0.00 | |
| 22 | 0 | 0 | 0 | 0 | 0 | 0 | 0 | 0 | 0 | 0 | 0.00 | |
| Average |  | | | | | | | | | | | 0.71±1.16 |
